# Iron Oxide and Gold Based Magneto-Plasmonic Nanostructures for Medical Applications: A Review

**DOI:** 10.3390/nano8030149

**Published:** 2018-03-07

**Authors:** Thi Thuy Nguyen, Fayna Mammeri, Souad Ammar

**Affiliations:** 1 Interfaces, Traitements, Organisation et Dynamique des Systèmes, ITODYS, UMR 7086, CNRS, Université Paris Diderot, Sorbonne Paris Cité, 15 rue Jean Antoine de Baïf, 75205 Paris, France; 2 Department of Advanced Materials Science and Nanotechnology, University of Science and Technology of Hanoi, Vietnam Academy of Science and Technology, 18 Hoang Quoc Viet, Hanoi, Vietnam

**Keywords:** magneto-plasmonic, nanocrystals, spherical, non-spherical, core-satellite, seed-mediated

## Abstract

Iron oxide and gold-based magneto-plasmonic nanostructures exhibit remarkable optical and superparamagnetic properties originating from their two different components. As a consequence, they have improved and broadened the application potential of nanomaterials in medicine. They can be used as multifunctional nanoprobes for magneto-plasmonic heating as well as for magnetic and optical imaging. They can also be used for magnetically assisted optical biosensing, to detect extreme traces of targeted bioanalytes. This review introduces the previous work on magneto-plasmonic hetero-nanostructures including: (i) their synthesis from simple “one-step” to complex “multi-step” routes, including seed-mediated and non-seed-mediated methods; and (ii) the characterization of their multifunctional features, with a special emphasis on the relationships between their synthesis conditions, their structures and their properties. It also focuses on the most important progress made with regard to their use in nanomedicine, keeping in mind the same aim, the correlation between their morphology—namely spherical and non-spherical, core-satellite and core-shell, and the desired applications.

## 1. Introduction

Iron oxide nanoparticles (NPs) of a maghemite (Fe_2.67_O_4_) or magnetite (Fe_3_O_4_) composition have demonstrated their strong potential for diagnostic and therapeutic applications due to their unique magnetic properties [1,2,3]. Indeed, these NPs can be used as contrast enhancement agents in magnetic resonance imaging (MRI). They can also generate heat under an external alternating magnetic field, making them valuable for magnetic hyperthermia therapy [4,5,6]. Besides, they can be guided and immobilized close to a specific biological target thanks to an external dc magnetic field for magnetically assisted drug delivery. Highly magnetized superparamagnetic NPs are a good choice for all these diagnostic and/or therapeutic uses. As high is the magnetization of these NPs, as high is their magnetic response to an external magnetic stimulus and as high is their efficiency in electromagnetically assisted nanomedicine.

Gold NPs exhibit also strong unique and tunable optical properties including Mie scattering, surface plasmonic resonance (SPR), surface-enhanced luminescence and surface Raman scattering. Moreover, gold surface is nontoxic and chemically stable under physiological conditions. It can be easily functionalized through thiol groups leading to Au-SH (gold-thiol) bio-conjugation for various ligands using immune targeting as biomarkers. Nowadays, they are used for high sensitivity and high resolution optical imaging [7,8]. Their multiplexing capabilities make them also valuable for bio-sensing [9,10,11,12,13]. More recently, they started to be employed for in vivo photothermal therapy [14,15,16,17,18], particularly when their SPR wavelength is in the near-infrared region (NIR), to avoid body tissues light scattering. 

Many attentions have been focused on the strategies for the fabrication of multifunctional nanostructures (NSs), combining ether the magnetic or the plasmonic properties of iron oxide or gold NPs, respectively, to the biological activities of selected biomolecules attached at their surface. For example, molecular functionalization of magnetic or plasmonic NPs by antibodies, proteins and/or organic ligands allows the production of nanohybrids exhibiting both physical and biological properties. This interest has also concerned the integration of magnetic NPs and other functional solids, like quantum dots (QDs) and noble metals (gold, silver…) [13,15,17,19,20,21] in single nano-objects, potentially stimulated by both magnetic and electrical fields, providing simultaneously magnetic and optical responses. Focusing on this kind of probes, a rapid overview of the relevant literature shows that they are commonly prepared by (i) coupling two particle populations, exhibiting either magnetic or optical properties, and embedding them in a same biocompatible polymer or ceramic matrix; (ii) coagulating both magnetic and optical, mainly plasmonic, NPs or (iii) performing a sequential growth of the two components, forming thus well-architected NSs of various morphologies (core-shell, raspberry, etc.) [13,22]. 

In the first chemical strategy, polymeric or ceramic matrix, which possesses colloidal stability and low cytotoxicity, constitutes the main component of the material carrier. It offers a protective layer from aggregation and/or oxidation to the embedded NPs. In addition, incorporation of molecular dyes and/or drugs inside the carriers provides advantages for a wide range of biomedical applications [23,24,25,26,27]. This kind of NSs is usually produced by the self-assembly (or sol-gel process) of polymer and silica wrap to preformed functional NPs, including iron oxide and gold particles. 

In the second strategy, the produced aggregates [28,29,30,31,32,33] including hollow aggregates, possess random and polydispersed NPs of iron oxide and plasmonic NPs, mainly gold. These structures are built thanks to a strong metal–oxide interaction at the interface of the two kinds of involved particles. Hollow ones provide high specific surface and low density [30,31,32,33], leading to an interest in the field of high drug-loading capacity. In addition, their plasmonic components exhibit not only a superior surface-enhanced Raman scattering (SERS) sensitivity, but also an excellent catalytic activity [28,29].

In the third strategy, the serial growth material processing is privileged. The competition between homogeneous and heterogeneous nucleation mechanisms represents the main issue affecting the morphology of the engineered NSs. In other words, starting from a seed phase is crucial to promote an exclusive growth of the new phase on the surface of the former. The promotion of heterogeneous nucleation while suppressing homogeneous one can be achieved by adjusting the reaction parameters such as the temperature, the precursor atomic ratio, the reaction time, the pH, among others. In these conditions and only in these conditions, uniformly distributed in size core-satellite or core-shell NSs can be produced. These sequentially grown Fe_3−*x*_O_4_ (*x* = 0 for magnetite and *x* = 0.33 for maghemite) on Au, or reversely Au on Fe_3−*x*_O_4_, NSs are very probably the most investigated multifunctional platforms for biomedicine. They have been naturally considered as dual heating sources as well as dual imaging probes. Beside their remarkable optical and magnetic properties provided by their plasmonic and superparamagnetic components, they offer the benefit of the control of their physical properties and then, their whole response to any external electromagnetic stimulus, through the control of their synthesis conditions. Indeed, the chemical composition, size and shape adjustment of each component achieved by the control of each synthesis parameter affects directly the plasmonic absorption wavelength and intensity of the built NSs as well as their total magnetization and their average blocking temperature value.

So, we propose in this review to summarize the recent achievements in the preparation of this last type of NSs, distinguishing the objects made from iron oxide cores surrounded by gold either as satellites or as a continuous shell and reversely, those made from gold cores surrounded by iron oxide either as satellites or as a continuous shell. 

Many chemical and physical routes have been proposed to prepare such NSs, but chemical deposition processes are certainly the most employed ones. They consist in direct deposition of seeds on preformed cores followed by a controlled growth of the seeds to form a more or less continuous coating around them. So, the main achievements of the literature in this research area are described hereafter, with a special emphasis on the most important progress made on their use in nanomedicine, with always the same aim, the correlation between their morphology and their properties. The review will be thus organized in three mains sections, a first one dedicated to the preparation of Fe_3−*x*_O_4_-Au (FANSs) and Au-Fe_3−*x*_O_4_ (AFNSs) NSs, respectively, a second one focusing on their magneto-plasmonic properties in relation to their structure and microstructure, and finally a third one describing their main use in biomedicine, in relation with their physical properties.

## 2. Structure and Synthesis of Magneto-Plasmonic Nanostructures Based on a Sequential Growth in Solution

### 2.1. Fe_3−x_O_4_-Au Nanostructures (FANSs)

The most described magneto-plasmonic NSs are the FANSs, consisting in a Fe_3−*x*_O_4_ single core or multicores on whose surface Au coating agents have been attached, allowing the growth of a continuous or discontinuous Au shell. FANSs can be spherical or non-spherical in shape (nanospheres, nanorods, nanoflowers, nanostars…). Their magnetic properties are mainly driven by the chemical composition (maghemite or magnetite), the size and the architecture of their core component (single core or multicores) while their plasmonic properties are mainly defined by the shape of their Au layer component. Interestingly, thanks to their gold surface that can be easily functionalized by thiol groups making FANSs conjugated to various biological molecules is easy. Gold surface reactivity toward various hydrophilic ligands and polymers allows also convenient surface functionalization of FANSs improving their biocompatibility and their aqueous colloidal stability. Moreover, the gold layer acts as a protection layer toward chemical oxidation, preserving, hence, the intrinsic properties of the core material, and as a diamagnetic layer, limiting magnetic mutual attraction and preventing from FANSs agglomeration and flocculation in a solution. 

FANSs are mainly used in scattering based imaging producing high sensitivity, high resolution and multiplexing capability. They are also used as contrast agent for MRI and heating sources for magnetic hyperthermia. 

#### 2.1.1. Different FANS Architectures

**Figure 1:**
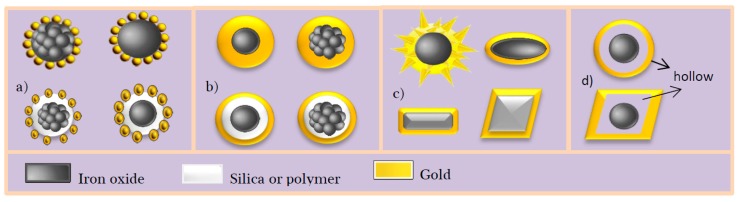
Main engineered structures of Fe_3−*x*_O_4_-Au Nanostructures (FANSs): (**a**) core-satellites; (**b**) spherical core-shell; (**c**) non-spherical core-shell and (**d**) hollow structures.

The different structures of FANSs can be listed according to the shape of Au shell and divided into 4 types: (a) core-satellites; (b) spherical core-shell; (c) non-spherical core-shell and (d) spherical and non-spherical hollow structures (Figure 1).

##### Fe_3−*x*_O_4_-Au Core-Satellite Structure 

Fe_3−*x*_O_4_-Au core-satellite structure is defined as a particle with a single or multi Fe_3−*x*_O_4_ cores surrounded by several Au small particles. The gold coating does not cover the whole Fe_3−*x*_O_4_ component surface, forming a satellite-type layer all around. The main advantage of such morphology is to provide a high gold specific area and to offer the possibility of a further functionalization of the uncovered Fe_3−*x*_O_4_ surface, eventually different from that engaged on Au surface. FANSs can also be employed as seeds to grow a complete Au layer, leading to Fe_3−*x*_O_4_-Au core-shell structures. 

##### Spherical Fe_3−*x*_O_4_-Au Core-Shell Structure

Fe_3−*x*_O_4_-Au spherical core-shell structure includes a single or multi Fe_3−*x*_O_4_ cores completely covered by a gold spherical shell. Compared to the core-satellite structure, the core-shell one exhibits lowest magnetization due to a higher diamagnetic contribution of gold, but an improved stability and biocompatibility in aqueous media. Indeed, the Fe_3−*x*_O_4_ cores are fully covered by Au, making them chemically inert, even in corrosive environments like biological ones. In addition, based on the Mie theory, the optical properties of these structures are quite different from those of individual Au spherical NPs. Therefore, by only varying the ratio between the Fe_3−*x*_O_4_ core size and the Au shell thickness, it becomes possible to easily tune the SPR gold response from visible to near-infrared region. 

##### Non-Spherical Fe_3−*x*_O_4_-Au Core-Shell Structure

Non-spherical Fe_3−*x*_O_4_-Au core-shell particles are composed by Fe_3−*x*_O_4_ single or multi cores surrounded by a non-spherical Au coating, like stars. As previously, the outer gold shell covers the whole Fe_3−*x*_O_4_ surface and prevents thus its further oxidation and corrosion. It also increases the stability and the biocompatibility of the resulting material. Additionally, the anisotropic shape of the gold shell leads to unusual optical features that are difficult to achieve using spherical one. Typically, a shift of the plasmonic absorption wavelength to higher values, matching very well with the spectral body windows, can be observed, improving thus significantly the efficiency of the designed structures for in vivo biomedical applications.

##### Fe_3−*x*_O_4_-Au Hollow Structure

Fe_3−*x*_O_4_-Au hollow structures are mainly based on Fe_3−*x*_O_4_ spheres surrounded by Au wall with a hollow interior, leading to large specific area and high reactivity. Among Fe_3−*x*_O_4_-Au core-shell nanoparticles, Au's hollow structure have indicated the attractive SPR feature which can be tuned from visible to NIR by varying the size, shape, and structure of the gold shell. These structures include integration both the magnetic properties and optical properties of iron oxide core and gold shells. In addition, the hollow interior provides high specific surface, low density and the possibility of physically entrapping drugs. Drugs may be loaded by encapsulation inside the hollow structure or by surface attachment where drugs are conjugated to the inner or outer gold surfaces using labile bonds.

#### 2.1.2. FANS Synthesis

**Figure 2:**
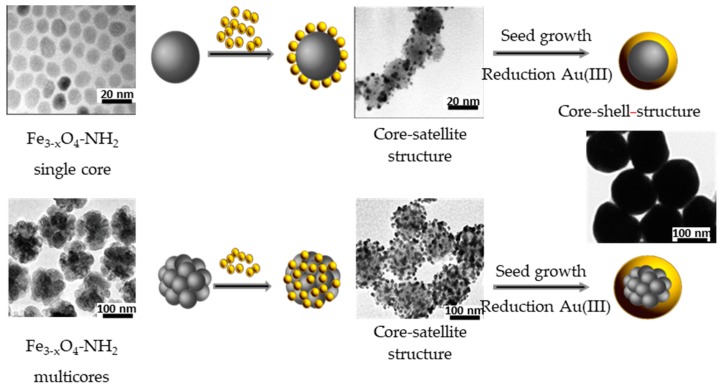
Schematic overview of the formation of core-satellite (top: reproduced with permission from [46]. American Chemical Society, 2005, and bottom: reproduced with permission from [47]. Royal Society of Chemistry, 2015) and core-shell spherical structure (representative TEM image reproduced with permission from [48], American Chemical Society, 2016).

There are various approaches to synthesize FANSs. One of the simplest consists of combining gold and iron oxide nanoparticles through an assembly process linking two separately prepared NPs. Au and Fe_3−*x*_O_4_ NPs are first functionalized by smart ligands, bearing in one side a group, which will be attached to the NP surface, and in the other side another functional group. For example, Au NPs attached through a thiol group to a ligand bearing negatively charged –CO_2_^−^ species, and Fe_3−*x*_O_4_ NPs attached through a diol group to a ligand bearing positively charged –NH_3_^+^ may interact electrostatically to form a robust Fe_3−*x*_O_4_-Au self-assembly. Negatively charged Au NPs (of 3–20 nm in diameter) can be easily synthesized in aqueous solution through the reduction of HAuCl_4_ by sodium citrate or NaBH_4_ [34,35], and subsequently electrostatically attached to Fe_3−*x*_O_4_ functionalized by positively charged amino groups. Fe_3−*x*_O_4_ NPs can be produced otherwise by thermal decomposition, aqueous co-precipitation, forced hydrolysis in polyol and solvothermal route. The former methods lead to the production of relatively small in size single crystals (<50 nm in diameter), while the latter allows the production of larger in size single and/or polycrystals (30–400 nm) [36,37,38,39,40,41,42]. These NPs can be then modified by grafting organosilanes, phosphonic acids, diol or carboxylate-based ligands or polymers bearing amino terminal groups that can interact with Au NPs (Figure 2). Typically, 3-aminopropyltriethoxysilane (APTES) [34,37], dopamine [42], polyethylene imine (PEI) [35,36,40,41], l-cysteine [38], poly (methyleneimine) [43], poly-(diallyldimethyl ammonium chloride) [44], cysteamine [45] have been extensively used for such a purpose.

**Figure 3:**
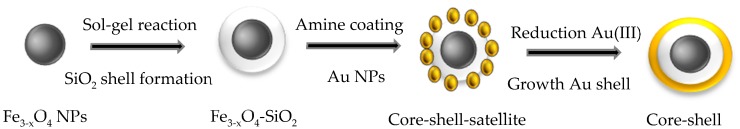
Schematic view of the formation of a silica layer around an iron oxide core before the deposition of gold satellites and shell by seed-mediated growth.

Alternatively, magnetic single or multi cores can be coated by a uniform silica shell, and then modified with an amine group [10] (Figure 3). 

**Figure 4:**
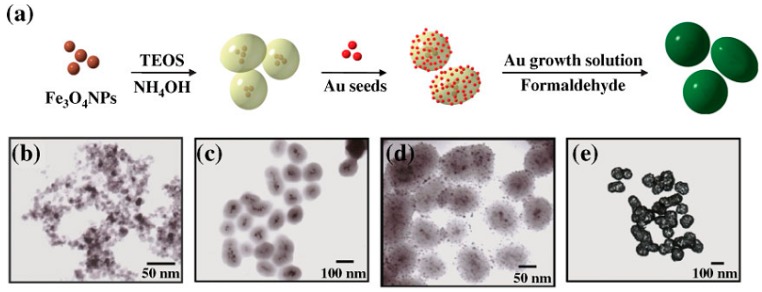
(**a**) Schematic representation of the preparation of Fe_3_O_4_-Au nanoeggs. TEM images of the (**b**) magnetic iron oxide NPs; (**c**) silica nanoeggs embedding magnetic iron oxide NPs (Fe_3_O_4_-SiO_2_); (**d**) gold nanoseeds bound to Fe_3_O_4_-SiO_2_ NPs (Fe_3_O_4_-SiO_2_-Au); and (**e**) Fe_3_O_4_-Au nanoeggs. Reproduced with permission from [49]. John Wiley and Sons, 2009.

The preparation of FANSs may also proceed by a chemical deposition process involving the direct reduction of dissolved Au(III) species onto the Fe_3−*x*_O_4_ surface according. In this case, two different chemical pathways may be followed. In a first one, the formed satellites act as nucleation points (seeds) for the subsequent continuous metallic layer growth, using formaldehydes [10,42], *N*,*N*-dimethylformamide (DMF) [40], NaBH_4_ [35], among others, as reducing agent. This method requires several steps but it allows the control of the Au shell morphology. Alternatively, iron oxide NPs can be first coated by silica, before the gold seeds attachment and the further continuous gold shell growth. Huang et al. [49] prepared, for instance, Fe_3_O_4_ multicores embedded in a SiO_2_ matrix by the Stӧber method (Figure 4). Then, they functionalized the outer silica layer by 3-aminopropyltrimethoxysilane (APTMS), which electrostatically interacted with negatively charged preformed gold NPs. At the end, they used the as-produced products as platforms for gold shell growth via an in-situ reduction of chloroauric acid precursor by formaldehyde reagent.

**Figure 5:**
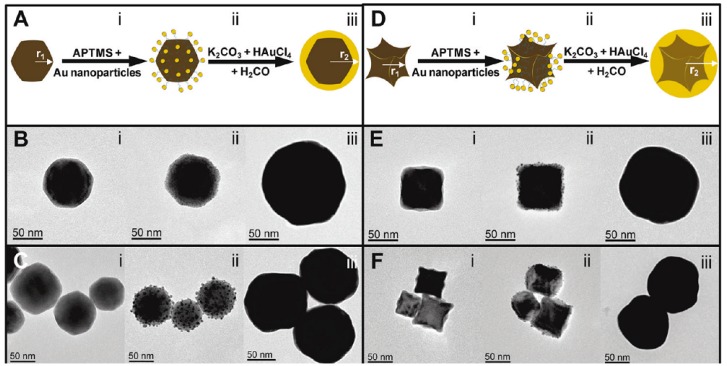
Schematic overview of the gold coating of (**A**–**C**) faceted and (**D**–**F**) tetracubic iron oxide cores. Representative TEM images of (i) the starting iron oxide cores; (ii) the decorated ones by Au precursors; and (iii) the coated ones by a continuous Au shell are given for illustration. Reproduced with permission from [50]. American Chemical Society, 2009.

A variety of shape and sizes of magnetic core NPs were synthesized by Halas et al. [36], using wustite FeO nanocrystals as precursors to form magnetic oxides of higher oxidation state, and to then grow a continuous gold shell layer all around. The coverage of the iron oxide surface by the gold shell was initiated by electrostatic interactions between the magnetic surface and the preformed negatively charged gold NPs, leading to a core-satellite architecture. Then, it was achieved by an in-situ reduction of auric cations at the surface of the gold seeds. At the end, whatever the morphology of the wustite precursors was, the morphology of the final core-shell particles was almost spherical (Figure 5). 

**Figure 6:**
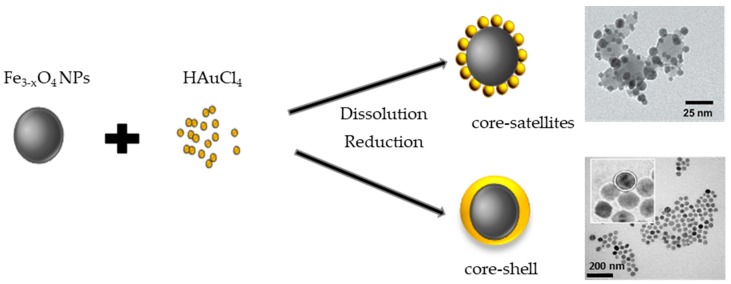
Schematic view of the formation of core-satellites. Reproduced with permission from [53]. Elsevier, 2009, and core-shell spherical structures via the so-called one step route. Reproduced with permission from [54]. John Wiley and Sons, 2015.

In the second route, called also one-step route, the Fe_3−*x*_O_4_ surface is directly coated by a gold layer [51,52]. This implies that Au atoms are directly deposited onto Fe_3−*x*_O_4_ through the reduction of Au(III) precursors (Figure 6). The basic principle for the formation of the gold shell is that Fe_3−*x*_O_4_ core surface must be appropriately charged to attract the dissolved molecular Au(III) precursors. This method can be accomplished in the absence of surfactant and completed in a one single step, but it does not allow a simple control of the final morphology of the built heterostructures. 

Very often, sodium citrate is used for reducing Au^3+^ into Au^0^ in the presence of Fe_3−*x*_O_4_ NPs [8,55]. In practice, the surface of Fe_3−*x*_O_4_ NPs was first modified by sodium citrate to improve their stabilization in water. Au atoms were deposited onto Fe_3−*x*_O_4_ surface through the reduction of chloroauric acid at the boiling temperature of the reaction solution [56]. Other reducing agents such as DMF [53], NaBH_4_ [57], hydroxylamine (NH_2_OH) [58] were also successfully used for the reaction. The thickness of Au shell was then controlled by changing the ratio between Fe_3−*x*_O_4_ NPs and Au precursors.

**Figure 7:**
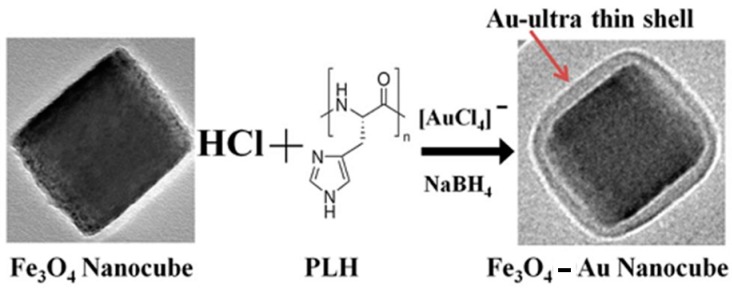
Schematic representation of the synthesis of Fe_3_O_4_-Au core–shell nanocubes, starting from monodisperse hydrophobic magnetic nanocrystals, coated with poly-l-histidine (PLH). The simultaneous addition of gold precursors and reducing reagents leads to the formation of a thin and continuous gold layer on the core nanocubes. Reproduced with permission from [59]. Royal Society of Chemistry, 2013.

To produce FANSs with anisotropic in shape Au satellites or shell, the previously described processing routes must be adapted, assuming that the spherical geometry corresponds obviously to the lowest energy shape. Non-spherical morphologies tend to become round with time, to reach the thermodynamical stability. Usually, non-spherical FANSs were produced by controlling experimental parameters like the reducing agent nature, the temperature and time reaction, the nature of gold and/or iron precursors, among others. This control must ensure an anisotropic Au growth onto an anisotropic in shape Fe_3−*x*_O_4_ core, while preserving the starting morphology, or induce an anisotropic Au growth onto an isotropic in shape Fe_3−*x*_O_4_ core, leading to a final anisotropic morphology. For example, Fe_3−*x*_O_4_ nanocubes were functionalized by the positively charged polypeptide, poly-l-histidine (PLH), through charge–charge interactions [59]. The positively charged surface supports AuCl_4_^−^ ions adsorption and then their subsequent reduction by NaBH_4_. Fe_3−*x*_O_4_, surface modified by imidazole groups, was also able to bind electrostatically to Au(III) ions, leading to a high Au^III^-surface packing density. A controlled reduction of these surface ions results into the formation of an ultra-thin Au shell, while preserving the starting cubic iron oxide core morphology (Figure 7).

**Figure 8:**
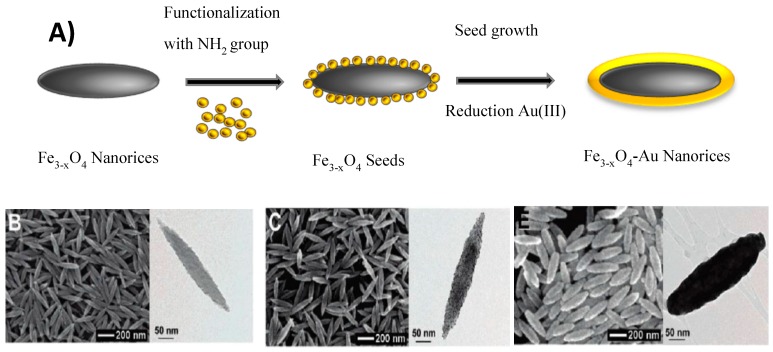
(**A**) Schematic view of the formation of non-spherical core-shell nanoparticles (NPs) starting from anisotropic in shape iron oxide cores. (**B**–**E**) Scanning electron microscopy (SEM) (**left**) and TEM (**right**) images of (**B**) hematite cores; (**C**) core-satellite particles used as seeds; (**E**) core-shell nanorice particles. Reproduced with permission from [60]. American Chemical Society, 2006.

Halas’ group also reported the synthesis of Fe_3−*x*_O_4_-Au core-shell nanorices, starting from anisotropic in-shape iron oxide core and using the seed-mediated method. Fe_3−*x*_O_4_ nanorices with a longitudinal diameter of 340 nm and a transverse diameter of 54 nm were prepared by heating FeCl_3_ and potassium dihydrogen phosphate. Their surfaces were then modified by NH_2_ groups using APTMS. Gold seeds adsorbed on their surface played the role of nucleation sites for the further growth of the gold shell. At the end, Au shells of 10–30 nm in thickness were formed around the Fe_3−*x*_O_4_ rices, thanks to the reduction of HAuCl_4_ by formaldehyde in the presence of the seeds. The formation of Au shell preserved the anisotropic shape of iron oxide cores, leading to plasmonic properties in NIR region (Figure 8). 

**Figure 9:**
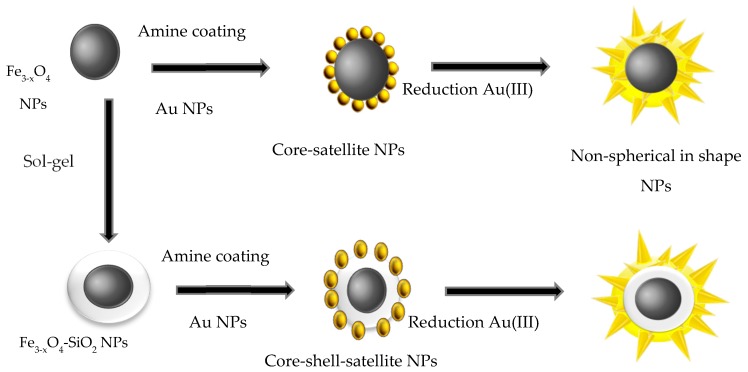
Schematic overview of the formation of non-spherical in shape core-shell structures via seed-mediated method.

Seed-mediated growth is the most common procedure to synthesize anisotropic in shape hetero-nanostructures starting from spherical Fe_3−*x*_O_4_ cores (Figure 9). In brief, Fe_3−*x*_O_4_-Au core-satellite structures were prepared and used as seeds for the anisotropic growth of the desired gold layer. Au seeds can be formed directly or indirectly on the iron oxide core surface. Alternatively, they can be formed on an intermediate layer of silica sandwiched between the core and Au seeds [61]. Finally, to grow the desired anisotropic in shape Au shell on the seeds, the choice of the reducing agent is of primary importance. It determines the formation rate of Au^0^ and affects thus the morphology of the final gold coating. Reducing agents such as hydroquinone [62,63,64] and *N*,*N*-dimethylformamide (DMF) [13,14,22] usually produce branched gold shell exhibiting strong absorption in the near infrared region. The addition of silver ions also promotes the growth of anisotropic Au shell [17,61]. The presence of silver ions on the gold surface prevents its further growth, favouring irregular shapes. The growth mechanism of anisotropic gold shell is the result of the interplay between the facet binding tendency of the stabilizing agents and the kinetically controlled growth. Besides, dynamic growth happens on all crystal facets, leading to the formation of spherical or near-spherical structures. By optimizing the synthesis conditions, driving kinetic differences in growth rate, atoms can be assembled along specific directions resulting in formation of anisotropic structures. Capping molecules adsorbed onto specific facets offer good conditions to enhance the crystal growth in certain directions and to avoid it in others. The interaction of different gold crystal facets with surfactants could physically drive their anisotropic growth. 

Kwizera et al. [48] synthesized 70–250 nm sized Fe_3−*x*_O_4_-Au heterostructures using a seed-mediated growth method. Newly formed gold atoms were added onto gold-seeded iron oxide octahedrons to lead to a gold shell. The evolution of the geometry of the shell was found to occur after the coalescence of gold seeds, which was achieved by controlling the amount of additive (silver nitrate) and/or specific reducing agent (ascorbic acid) in the growing solution. Experimental results were found to be in good agreement with the data provided by molecular modelling, demonstrating the intimate roles of thermodynamic and kinetic parameters in the shape-controlled synthesis of nanopopcorns or nanostars. 

**Figure 10:**
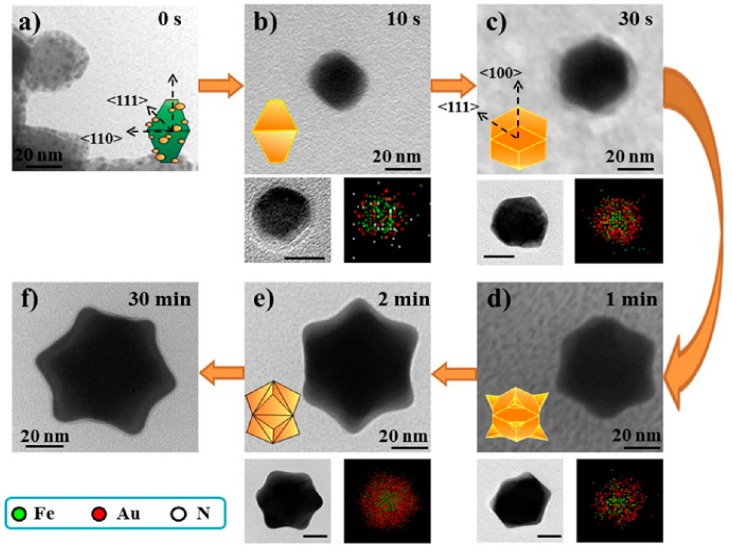
TEM images of Fe_3_O_4_-Au NPs and their elemental analysis (spot-mapping) for different reaction times: (**a**) 0; (**b**) 10 s; (**c**) 30 s; (**d**) 1 min; (**e**) 2 min; and (**f**) 30 min. Iron appears in green, gold in red and nitrogen in white. Reproduced with permission from [65]. American Chemical Society, 2014.

Li et al. [65] prepared truncated octahedral Fe_3_O_4_ cores coated with a trisoctahedral Au shell. In practice, they attached poly-l-lysine on the surface of the Fe_3_O_4_ cores, to form an intermediate layer, and then submitted the resulting hybrids to a subsequent gold seeded growth, for different reaction times as illustrated in Figure 10. 

Concerning the synthesis of the last type of FANS architecture, namely hollow Fe_3−*x*_O_4_-Au core-shell NS, a few numbers of reports exists in the relevant literature. They are usually prepared starting from multifunctional multilayer of Fe_3−*x*_O_4_-silica-Au [10,49] or Fe_3−*x*_O_4_-polymer-Au core-shell NSs [38,44,45], and performing etching treatment to remove the intermediate layer of silica or polymer sandwiched between the Fe_3−*x*_O_4_ core and Au shell to make hollow interior. In practice, iron oxide NPs are functionalized to make their coating by silica or polymer possible. Gold is then prepared by a series of growth of Au atom onto Fe_3−*x*_O_4_ surface through the reduction of chloroauric acid. After that, the interface between iron oxide core and Au shell is removed to produce hollow structures. Urries et al. [66] reported Fe_3−*x*_O_4_-Au hollow structures for magnetic resonance imaging, drug delivery and NIR hyperthermia application. Hollow or semi-hollow interiors have been successfully synthesized and their physico-chemical characteristics have been investigated. The resulting NPs maintain the magnetic and optical properties of the iron oxide core and Au shell respectively. By controlling the concentration of the etching agent and the etching time it is possible to remove the interior silica while preserving the inner magnetic cores and the SPR of the Au shell. The hollow interior can be used to store drugs or molecules of interest in the partially etched nanoshells. The final construct has potential as a theranostic platform, combining two therapeutic possibilities (drug delivery and NIR hyperthermia) and imaging capabilities in MRI applications.

### 2.2. Au-Fe_3−x_O_4_ Nanostructures (AFNSs)

**Figure 11:**
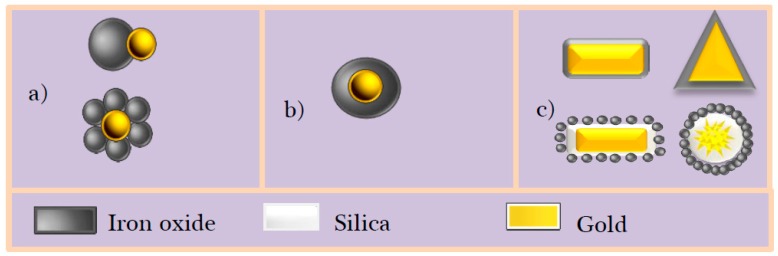
Some structures of Au-Fe_3−*x*_O_4_ Nanostructures (AFNSs): (**a**) dumbbells and core-satellites; (**b**) spherical core-shell and (**c**) non-spherical core-shell.

AFNSs are defined as NPs constituted by a single Au core or multiple Au cores, surrounded by a Fe_3−*x*_O_4_ coating. The structure of the Fe_3−x_O_4_ coating can be consistent with a single NP, satellites, flower-like structure or smooth thin layer leading to different AFNS architectures. Three main classes were thus defined: Au-Fe_3−*x*_O_4_ dumbbells, Au-Fe_3−*x*_O_4_ core-satellites and Au-Fe_3−*x*_O_4_ core-shell particles (Figure 11). Compared to FANSs, AFNSs may present interesting magnetic features, in relation (i) with the spin alignment at the interface between the non-magnetic gold matter and the magnetic iron oxide one; and (ii) with the dipolar interactions between the Fe_3−*x*_O_4_ coating from one AFNS to another. These features are expected to affect the response of AFNSs to an applied magnetic field, which in turn must change their behaviour during MRI or hyperthermia assays, compared to free Fe_3−*x*_O_4_ nanocrystals.

#### 2.2.1. Different Types of AFNSs

##### Au-Fe_3−*x*_O_4_ Dumbbell Structure 

These structures require a single Au NP and a single Fe_3−*x*_O_4_ NP linked by an interfacial bond. Fe_3−*x*_O_4_ may grow on a specific Au crystallographic plane or can be chemically attached on it, through a coupling agent, the other gold surface sites being blocked by ligands with zero affinity to Fe_3−*x*_O_4_. These structures possess a single plasmonic response. Moreover, their Janus-type bifunctional feature (metal and oxide) makes their surface adapted for different functionalization approaches, facilitating the attachment of various biological molecules, used for in vitro and in vivo biotargeting strategies.

##### Au-Fe_3−*x*_O_4_ Core-Satellite Structure

In these structures, a single and spherical Au core is surrounded by many Fe_3−*x*_O_4_ small particles, forming a discontinuous Fe_3−*x*_O_4_ coating. The main advantage of these NSs is the high surface area provided by the satellite layer, which can be, for instance, useful for chemical catalytic application. Through the integration of two functional components and two surface types, the engineered NSs keep the individual functions of the core and the shell, as well as they impart cooperative properties. Non-spherical Au core, on which small isotropic in shape Fe_3−*x*_O_4_ particles are distributed as satellites, forms also Au-Fe_3−*x*_O_4_ core-satellite structure.

##### Spherical Au-Fe_3−*x*_O_4_ Core-Shell Structure 

These particles consist mainly of a single Au spherical core, surrounded by a dense and continuous Fe_3−*x*_O_4_ shell. The surface of the gold core is absolutely inactivated but it keeps its optical properties. The advantage of this structure is to exhibit higher magnetization and higher blocking temperature compared to those of the core-satellite structures.

##### Non-Spherical Au-Fe_3−*x*_O_4_ Core-Shell Structure

In this case, the Au core is still wholly surrounded by Fe_3−*x*_O_4_ shell; however, its initial morphology is not spherical but looks like more to rods, flowers, stars… The Fe_3−*x*_O_4_ coating covers fully its surface. Compared with the spherical Au-Fe_3−*x*_O_4_ core-shell structures, the non-spherical ones possess an anisotropic in shape plasmonic core surrounded by a phase presenting a high refractive index and dielectric constant. As a consequence, their optical absorption bands are much more broadened toward the NIR region.

#### 2.2.2. Synthesis of AFNSs

The preparation of AFNSs has been mainly developed around the chemical deposition processes and has been often achieved by sequential growths on Au surface. As for FANSs, there are two main processing approaches, the one-pot and the two-steps or modified seed-growth method. 

**Figure 12:**
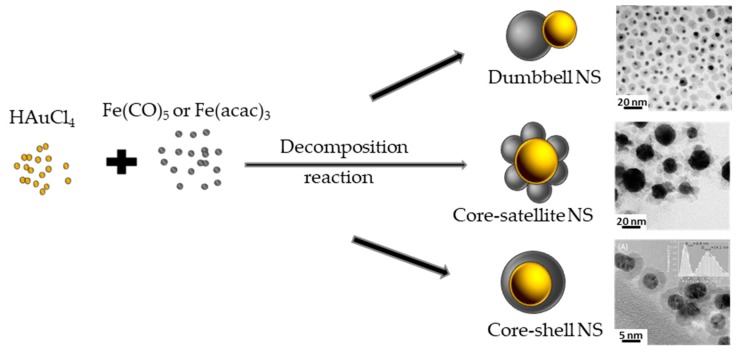
Schematic representation of the one-pot formation of Au-Fe_3_O_4_ dumbbell, core-satellite core-shell NSs. Representative TEM image of such NSs, reproduced with permission from [69]. Elsevier, 2012, reproduced with permission from [73]. Elsevier, 2016 and reproduced with permission from [74], are given for illustration.

The one pot method consists in mixing all precursors into the same reaction solvent and decomposing Au and Fe precursors during a heating processing to form the desired Au-Fe_3−*x*_O_4_ NSs. Typically, HAuCl_4_ was introduced in the reaction mixture at the beginning of the reaction [67,68,69,70,71] or during the heating step. Yu et al. [72] injected a solution of Au(III) chloride in toluene into a fresh Fe(CO)_5_ organic solution in the presence of oleic acid and oleylamine, and heated the mixture up to 310 °C. The size of the produced Au NPs was controlled by the choice of the injection temperature. An injection of Au precursor at 180 °C, provided 6 nm and almost spherical sized Au particles, while an injection at 120 °C resulted in 3 nm sized Au ones. Sheng et al. [69] also fabricated Au-Fe_3_O_4_ dumbbell NSs based on epitaxial growth of Fe_3_O_4_ onto Au, but they proceeded almost equivalently. They first formed Au particles at 140 °C by adding HAuCl_4_ into a solution containing oleic acid, oleylamine, 1,2-hexadecanediol and Fe(CO)_5_. Then, they increased the reaction temperature at a higher temperature to decompose Fe(CO)_5_. The formed Fe-based solutes started to grow on Au surface, leading to Au-Fe_3_O_4_ dumbbells with a size varying between 5 and 12 nm, just by adjusting the precursor ratio between Fe(CO)_5_ and HAuCl_4_ (Figure 12). 

Au-Fe_3_O_4_ core-satellite NSs were also prepared using the one-pot method, mixing HAuCl_4_ and Fe(acac)_3_ in triethyleneglycol (TREG) solvent [11]. TREG played the role of the solvent, reducing agent and capping ligand. The resulting NSs consisted of 80 nm sized almost isotropic in shape Au core decorated with very small iron oxide particles. 

Felix et al. [74] reported the preparation of spherical Au-Fe_3_O_4_ core-shell NSs using thermal decomposition method. They mixed both ferric acetylacetonate and auric acetate salts in 1-octadecene and 1,2-hexadecanediol in presence of oleylamine and oleic acid surfactants. They heated the reaction medium first up to 120 °C (for 30 min) and then up to 260 °C to produce the desired microstructure, made of a gold core of 7 nm in diameter and a magnetite shell of 3.5 nm in thickness.

**Figure 13:**
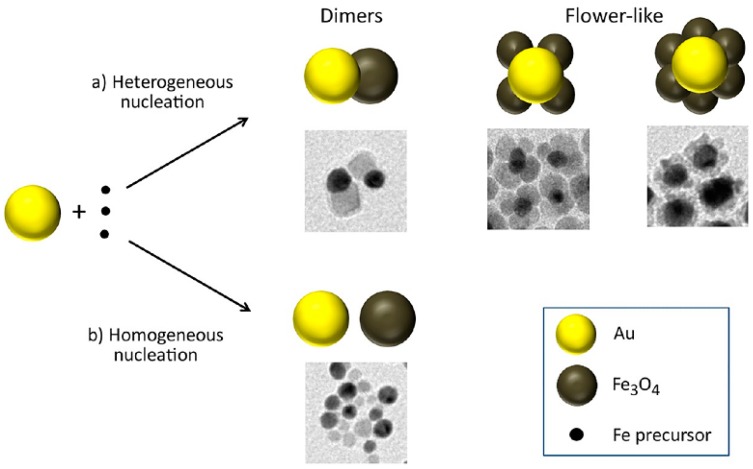
Schematic representation of the possible pathways of the seeded-growth reaction, starting from a mixture of Au nanocystals and molecules of iron precursor. (**a**) Heterogeneous nucleation of iron oxide, with the formation of either dimers or flower; (**b**) Homogeneous nucleation of iron oxide, with the consequent formation of isolated iron oxide particles. Reproduced with permission from [78]. American Chemical Society, 2017.

The two-steps method, called also modified seeded-growth, is like the seeding growth described previously for FANSs. In this approach, pre-synthesized Au NPs were used as seeds and were introduced into the reaction mixture, containing iron precursors. The growth of Fe_3−*x*_O_4_ onto Au surfaces occurred after the thermal decomposition of the iron precursors [69,75,76,77]. The growth of Fe_3−*x*_O_4_ depends on many parameters including the nature and the concentration of the iron precursor, the nature and concentration of the surfactants, the solvent polarity, the seed-to-precursor ratio, the temperature rate, the time for each heating plateau, etc. The polarity of solvents is a key parameter [78] since in a non-polar solvent, Au NPs are more negatively charged, preventing iron oxide nucleation and/or growth on their surface, after iron precursor, mainly Fe(CO)_5_, decomposition. As a result, a single site nucleation was obtained per Au particle to form dumbbell NSs. In polar solvent, the total negative charge of Au NPs decreases, permitting a multiple site nucleation of Fe_3−*x*_O_4_ on their surface and leading thus to the formation of core-shell structures (Figure 13).

**Figure 14:**
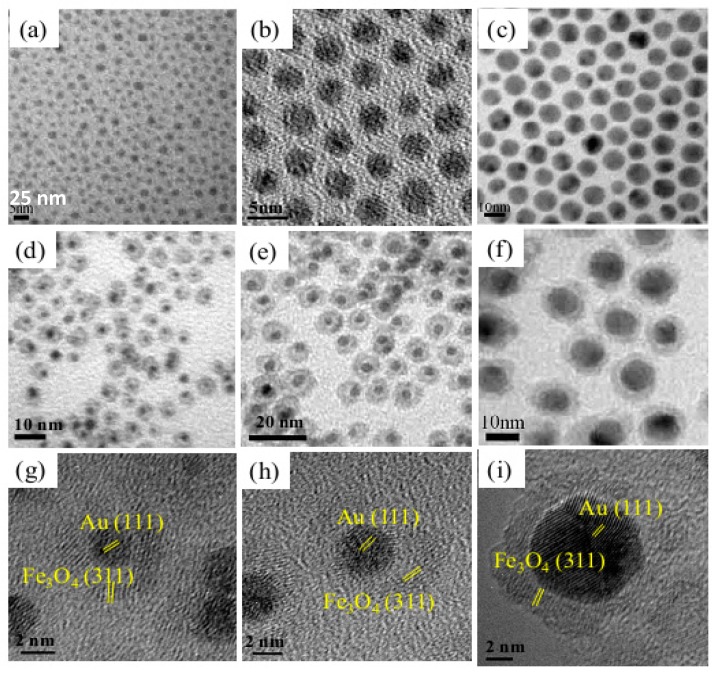
TEM images of Au NPs of (**a**) 2.5; (**b**) 4 and (**c**) 10 nm and the corresponding Au-Fe_3_O_4_ yolk-shell NSs starting from (**d**) 2.5; (**e**) 4 and (**f**) 10 nm sized Au cores. High resolution micrographs of Au-Fe_3_O_4_ yolk-shell NSs prepared starting from Au cores of (**g**) 2.5; (**h**) 4 and (**i**) 10 nm in size. Reproduced with permission from [79]. American Chemical Society, 2017.

Fantechi et al. [78] synthesized well-defined AFNSs starting from preformed Au seeds of 10 nm in diameter. By varying the nature of the iron precursor, namely Fe(CO)_5_ or Fe(acac)_3_, they observed that the latter is much more sensitive to the variation of the synthesis parameters than the former. So, its thermal decomposition may lead to both heterogeneous and homogeneous Fe_3_O_4_ nucleation and growth, depending on the reaction temperature and the solvent nature. Jiang et al. [76] prepared successfully Au-Fe_3_O_4_ dumbbells through the sequential decomposition of Fe(CO)_5_ in the presence of Au cores of various sizes (2.5, 4, 7 and 10 nm). They showed that the final morphology of the produced AFNSs can be controlled just by varying the gold seed size. Lin et al. [79] obtained the same results, using similar operating conditions (Figure 14). 

Non-spherical Au-Fe_3−*x*_O_4_ core-shell NSs have been also produced by one pot method and sequential growth. Most of the published works described the preparation of hetero-nanostructures based on anisotropic in shape Au core. Anisotropic Fe_3−*x*_O_4_ shells have been rarely reported. The preparation of non-spherical Au-Fe_3_O_4_ core-satellite NPs by one-pot method has been reported by Yu et al. [71]. They consist of gold nanotriangles, of about 280 nm in diameter, decorated by spherical superparamagnetic iron oxide NPs of 5 nm in diameter. These nanotriangles exhibited SPR in the NIR region (800 nm).

**Figure 15:**
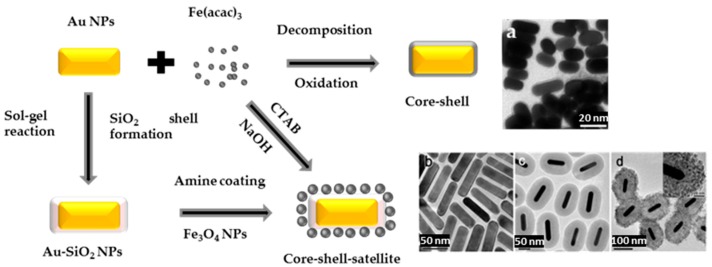
Non-spherical Au-Fe_3_O_4_ core-satellite NSs starting from Au nanorods. (**a**) TEM images reproduced with permission from [85]. Springer Nature, 2012. While (**b**–**d**) ones reproduced with permission from [84]. American Chemical Society, 2016.

Non-spherical Au-Fe_3−*x*_O_4_ core-satellite NSs can be assembled through electrostatic interactions, by adsorbing Fe_3−*x*_O_4_ nanocrystals at the surface of Au rods [80,81,82]. Uniform and smooth Fe_3−*x*_O_4_ shell coating was also achieved around Au nanorods, by directly reacting Fe(acac)_3_ on CTAB capped Au surface in aqueous solution at room temperature [83] (Figure 15). Au nanorods were also coated by silica [84], to enhance their colloidal stability in ethanol, before growing Fe_3−*x*_O_4_ by thermal decomposition of Fe(acac)_3_ at 290 °C (Figure 15). In this case, the intermediate silica layer plays the role of a bridge between the gold and iron oxide crystals. It also preserves the plasmonic feature of Au particles and makes Fe_3−*x*_O_4_ uniformly distributed. 

**Figure 16:**
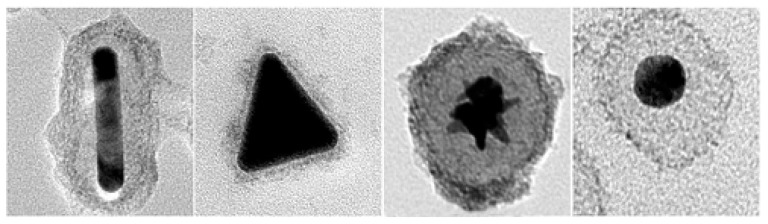
Some non-spherical AFNSs built using a PPy adhesion layer. Reproduced with permission from [86]. Springer Nature, 2016.

Yang et al. [86] reported the use of polypyrrole (PPy) as a thin mid cohesive layer between Au core and Fe_3−*x*_O_4_ shell (Figure 16), to encapsulate anisotropic in shape gold NPs, like nanorods, nanotriangles and nanostars. Then, many iron oxide nanocrystals were formed on the surface of Au-PPy nanohybrids. The resulting Au-PPy-Fe_3−*x*_O_4_ NSs showed excellent NIR photothermal conversion efficiency, biocompatibility, making them particularly appropriated as theranostic agents for magnetic resonance imaging and photothermal therapy.

## 3. Magneto-Plasmonic Properties of FANSs and AFNSs

### 3.1. Optical Properties

**Figure 17:**
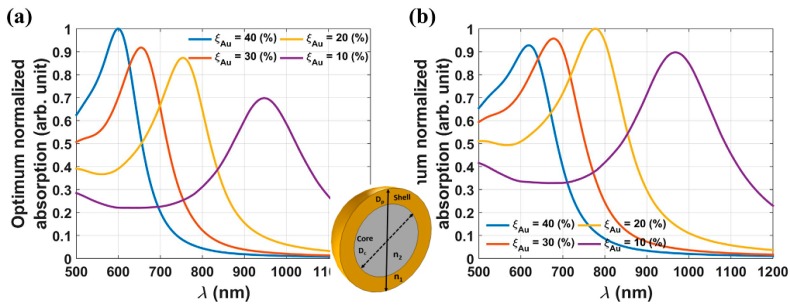
Parametric analysis of optimum normalized absorption vs. *λ* for two different sized Fe_3_O_4_@Au NPS with various shell-to-radius ratios *ξ*_Au_ (**a**) D_p_ = 60 nm, (**b**) D_p_ = 100 nm. Reproduced with permission from [87].

The optical properties of gold NPs are related to the interaction with light of their free conductive electrons on the metal surface. It is known as a surface plasmon resonance (SPR) that causes enhanced absorption and scattering intensities at the plasmon resonance wavelength. The intensity and position of SPR can be controlled by the size, the shape and the dielectric constant of the surrounding medium. SPR is slightly red-shift with increasing of size (Figure 17). 

Mie theory was used to predict the optical spectra of core-shell structured FANSs as a function of their chemical composition, size and shape. It is important to optimize the optical absorption for a large panel of bio-applications. Applying Mie theory, the optical spectra of such hetero-structures were calculated for two different particle sizes, 60 and 100 nm, and various ratios between the shell thickness and the core radius, 10%, 20%, 30%, and 40%. The obtained spectra showed that the plasmonic absorption band is red-shifted as the ratio decreased [87]. This feature was confirmed experimentally by Canet-Ferrer et al. [88], who evidenced that the extinction spectra of Fe_3_O_4_-Au core-shell NSs of 5 nm in diameter, exhibited the highest absorption wavelength (around 700 nm) when the gold thickness was the thinnest (about 1 nm). 

Theoretically, to obtain spherical core-shell structured FANSs with a plasmonic absorption over 800 nm, the core needs to be larger than 100 nm for a shell thickness of about 5 nm.

**Figure 18:**
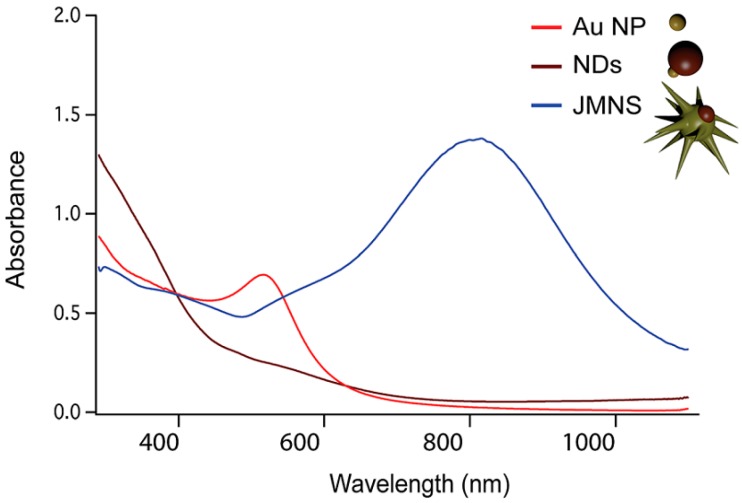
UV-Vis spectra comparing the absorbance of spherical Au NPs (in red), nanodumbbells (in brown) and nanodumbbell and star-shaped (in blue) FANS counterparts. Reproduced with permission from [22]. Royal Society of Chemistry, 2016.

Another way to shift the plasmonic absorption toward NIR region is to change the NS shape. For example, star-shaped core-satellite structured FANSs exhibit an absorption peak much more red-shifted than spherical ones, due to the sharp tip of gold crystals. These results are well-illustrated in Figure 18 where the UV-Vis-NIR absorption spectra of three kinds of gold-based particles are plotted. The plasmonic absorption of spherical Au NPs was found to be about 520 nm, while that of their nanodumbbell (NDs) and nanodumbbell and star-shaped (called JMNSs) FANS counterparts were red-shifted to 560 nm and 800 nm, respectively. 

**Figure 19:**
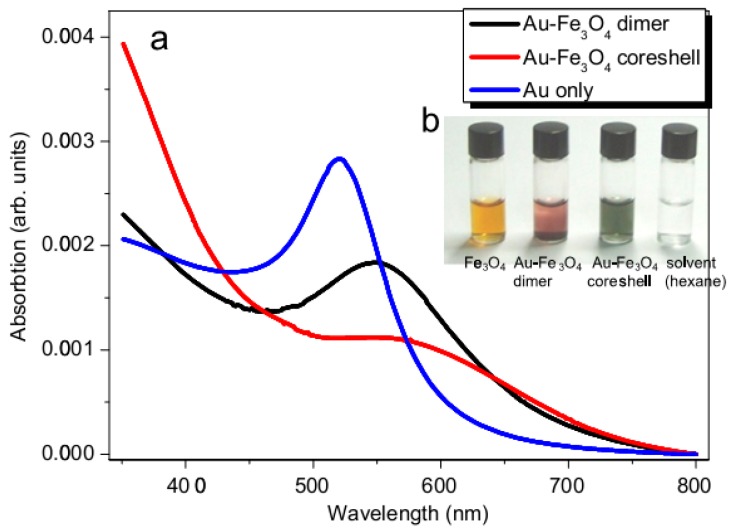
(**a**) Optical absorption spectra of ~8 nm sized Au, Au-Fe_3_O_4_ dimer and core-shell nanoparticles; and (**b**) image of Fe_3_O_4_ and Au-Fe_3_O_4_ NPs suspensions in hexane. Reproduced with permission from [70]. Elsevier, 2012.

SPR of AFNSs is also red-shifted as a consequence of the refractive index increase on the gold surrounding media [69,73,76,89]. For instance, Umut et al. [55] compared the absorption spectra of Au-Fe_3_O_4_ dumbbell and core–shell NSs, constituted of an approximatively 8 nm sized Au core. They observed that, whereas the spectrum of Au single core particles exhibits an absorption at around 520 nm, those of the engineered Au-Fe_3_O_4_ NSs exhibited a peak sifted to higher wavelengths (Figure 19). This shift was much more important for the core-shell architecture due to the high refractive index of the iron oxide coating [71]. 

### 3.2. Magnetic Properties

The magnetic properties of both FANSs and AFNSs are mainly driven by those of their magnetic component, depending on their size, composition and structure. For example, if the magnetic core size is below a critical size, the Fe_3−*x*_O_4_ crystals act as single magnetic domains, with a remanence and coercivity at room temperature. If their size decreases down to another limit, called the superparamagnetic limit, the thermal energy becomes larger than their magnetic anisotropic energy leading to a spontaneous reversal of their magnetization, with zero coercivity and remanence. This behaviour is also characterized by a net irreversibility between the thermal variation of the magnetic susceptibility, when measured in field cooling (FC) conditions and zero field cooling (ZFC) ones. In general, all the investigated gold and iron oxide based-heterostructures in the literature present a superparamagnetic behaviour.

**Figure 20:**
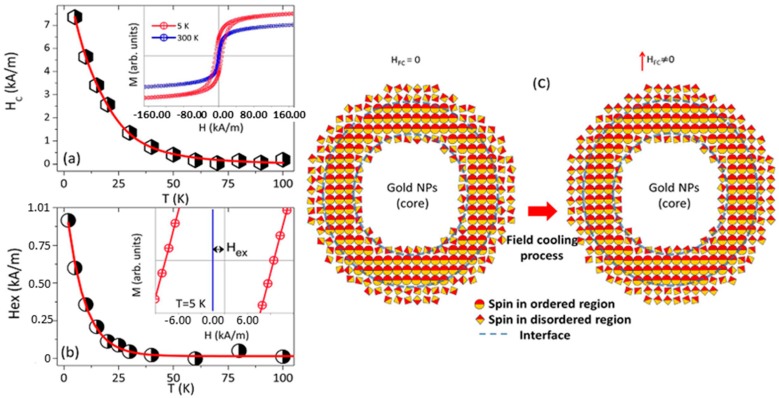
(**a**) Temperature dependence of the coercive field of the Au-Fe_3_O_4_ core-shell NP based fluid sample. The inset shows the hysteresis loops of the investigated AFNSs, as measured at 5 K and 300 K; (**b**) The temperature dependence of the exchanged bias field, for field cooling of 2 T. The inset illustrates the magnetization hysteresis loop shift at 5 K; (**c**) Schematic representations of the different magnetic regions in one particle and the effect produced by the cooling in a magnetic field. Reproduced with permission from [74].

The magnetic properties of FANSs and AFNSs are also significantly dependent on the surface or interface structure of their iron oxide component, its size and its interactions (dipolar or by exchange) with other iron oxide components. Indeed, the interface region between the iron oxide and gold includes broken bonds, which may generate randomness and/or frustration in the exchange interaction between the outer magnetic cations and the inner ones [90]. In some cases, an exchange bias feature can be observed as illustrated in Figure 20, with the appearance of a small shift, along the field axis, of the hysteresis loop recorded in FC conditions by comparison to that recorded in ZFC ones [74]. The iron oxide crystal size reduction leads also to surface iron cation spin disorder, which affects the total magnetic behaviour of the Fe_3−*x*_O_4_ matter, decreasing the whole magnetization and increasing the total anisotropy.

**Figure 21:**
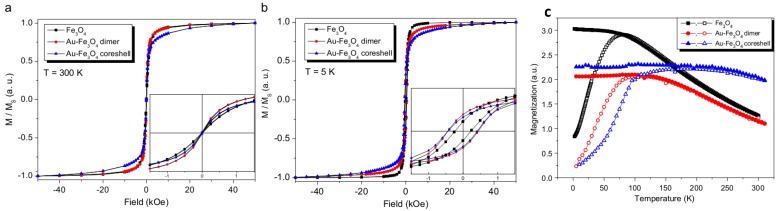
M/M_s_ hysteresis loops measured at 300 K (**a**) and 5 K (**b**) on powdered Fe_3_O_4_, dimer and core-shell Au-Fe_3_O_4_ NPs. ZFC (hollow symbols) and FC (solid symbols) magnetization measurements of the powder samples for an applied magnetic field H = 50 Oe (**c**). Reproduced with permission from [70]. Elsevier, 2012.

Recently, Felix et al. [74] reported the structural and magnetic properties of AFNSs based on a 6.9 ± 1.0 nm in diameter Au core and a 3.5 nm in thickness Fe_3_O_4_ shell. They observed that their hetero-nanostructures display a blocked state at temperatures lower than T_B_ = 59 K and a superparamagnetic one at temperatures higher than T_B_. They also evidenced an exchange bias feature when cooling their samples down to 40 K under an external magnetic field, as a consequence of the interaction between the spins located in the magnetically disordered regions (in the inner and outer surface of the Fe_3_O_4_ shell) and spins located in the ordered region of the Fe_3_O_4_ shell. They clearly measured a non-zero exchange field H_EX_, which characterizes the FC-hysteresis loop shift; they also observed a net anisotropy increase compared to bare Fe_3_O_4_ NPs. This increase of the magnetic anisotropy was also reported by other groups. A low temperature coercivity increase as well as a blocking temperature increase were observed by Umut et al. even for dumbbell AFNSs (Figure 21) [70].

An important feature of these heterostructures, which is poorly described in the literature, is the modulation of the strength of their mutual dipolar interactions, just by varying their final morphology. Indeed, AFNSs are expected to suffer much more from these interactions than FANSs, since the mutual distance between the magnetic components of two adjacent AFNSs is reduced compared to that between two adjacent FANSs. But in all the cases, these interactions can be significantly reduced when AFNSs (and also FANSs) are dispersed in aqueous media as stable colloids.

Finally replacing in FANSs and AFNSs, the iron oxide single core component by a multicores one, usually allows to increase the magnetization as well as the blocking temperature (polycrystals much more than nanoclusters), which can be particularly useful for magnetic hyperthermia applications [14]. Indeed, the specific absorption rate SAR, which defines the efficiency of a magnetic particle to be used as heating probes under an alternating magnetic field, depends directly on the square of the saturation magnetization [91,92,93]. The higher the magnetization, the higher the SAR value. 

## 4. Application of Magneto-Plasmonic Hetero-Nanostructures

### 4.1. Multimodal Imaging

Magneto-plasmonic nanostructures have been considered for multimodal imaging. MRI has been known as a non-invasive imaging to obtain highly contrasted images of any part of the body by applying external magnetic fields. The non-invasive feature relies on the different water content to distinguish between diseased tissues and healthy ones. Proton nuclear spins align in the direction of the applied DC magnetic field. Under a perpendicular Radio Frequency (RF) pulse, aligned protons are perturbed and return to their original state at the end of the pulse. There are two relaxation processes: the longitudinal relaxation (T1-recovery) and the transverse relaxation (T2-decay), which generate MR images. Superparamagnetic NPs, like iron oxide NPs, enriched in the tissue can provide MR contrast enhancement by shortening mainly T2 relaxation time of its surrounding water protons. They can be thus used as negative contrast agents for T2-weighted MRI.

Optical imaging is also a non-invasive diagnostic method. It is based on the use of light nanosources, which emit enough transparent light to be able to cross body tissues, with wavelengths ranging in the body spectral window, typically in the NIR region. Plasmonic NPs, like gold NPs, exhibit strong unique and tunable optical properties within the visible to NIR spectral region, and can play the role of nanosources. Besides, gold is characterized by a high atomic number, and thus, a high electronic density, making these NPs also suitable to be used in dark field microscopy (DFM), optical coherence tomography (OCT), computed tomography (CT) and photoacoustic (PA) imaging. 

Using magneto-plasmonic nanostructures for multimodal imaging, each component possesses featured strengths and weaknesses. The integration of many modalities in a single object is expected to give comprehensive information for diagnostic and treatments of a given disease, like cancers. For example, MRI is a useful imaging tool for the whole-body diffusion-weighted imaging in disease diagnosis. It is a technique with a high spatial resolution, with no tissue penetration limit, but it suffers from low sensitivity. CT is well known as one of the most common imaging modality, with high resolution and detailed 3D visual structure of tissues. However, CT imaging is low sensitive for low density. The integration of MR and CT provides more accurate diagnoses [94]. Hou et al. [95] reported a simple method for synthesizing monodisperse Fe_3_O_4_-SiO_2_-Au-PEG FANSs for coupled CT and MR imaging. The as-obtained NSs were composed by a superparamagnetic magnetite inner core, coated with a silica mid-layer and then with an outer gold nanoshell, itself covered by hydrophilic polyethyleneglycol. They exhibited a uniform size distribution with an average diameter of less than 100 nm. Zhu et al. [96] reported the fabrication of Au-Fe_3_O_4_ AFNSs for in vivo dual CT and MR imaging. They demonstrated that the Au-Fe_3_O_4_ NSs can give detailed structures of targeted organs, through their visualization by CT and by MRI. For instance, they succeeded to visualize simultaneously the right ventricle and the liver of a rabbit by CT and MR imaging. Li et al. [97] developed a simple one-pot hydrothermal approach to synthesize Fe_3_O_4_-Au core-shell structured FANSs and tested them for MR and CT imaging. They evidenced that the produced structures have a relatively high r_2_ relaxivity (146.07 mM^−1^ s^−1^) and a good X-ray attenuation property. So, they successfully used them as in vivo contrast agents for liver magnetic imaging and liver and aorta CT imaging, on a small mammal model (mouse). Wang et al. [98] reported a novel ideal AFNS multimodal contrast agents by assembling gold nanocages, functionalized by thiols, and ultra-small iron oxide nanocrystals for simultaneous T_1_–T_2_ dual MRI and CT contrast imaging. These probes exhibited a small average size, a low aggregation and an excellent biocompatibility. Their Au nanocages provided them a strong X-ray attenuation property for CT imaging, while their ultra-small Fe_3−*x*_O_4_ NPs made them able to give enhanced signal of T_1_- and T_2_-weighted MRI (r_1_ = 6.263 mM^−1^ s^−1^, r_2_ = 28.117 mM^−1^ s^−1^). Moreover, in vitro and in vivo studies revealed that they present a long-term circulation time, renal clearance and outstanding capability of selective accumulation in tumours, which is absolutely required for any valuable exogenous imaging probe.

**Figure 22:**
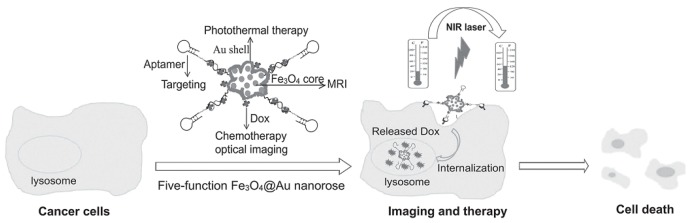
Schematic representation of five-function Fe_3_O_4_-Au nanorose for cancer cell targeting, MR and optical imaging, photothermal and chemotherapy. Sgc8 aptamers are conjugated to the surface of the nanoroses for targeting of CCRF-CEM malignant cells. Anticancer drug (Doxorubicine) is specifically delivered into cancer cells for chemotherapy. Reproduced with permission from [64]. John Wiley and Sons, 2014.

Compared to spherical AFNSs, those presenting an anisotropic shape, e.g., nanostars, nanorods, etc., are much more pursued for biomedical applications, due to their strong SPR in NIR region. They allow in vivo optical imaging and photothermal therapy without any body limits. Many achievements in the development of multimodal MRI and optical imaging or MRI and photothermal therapy using AFNSs have been reported [17,64,99,100,101] (Figure 22). Larson et al. [102] reported the use of gold-coated iron oxide NPs for combined molecular specific MRI and optical imaging to help guided-laser therapy of cancer cells. The gold shell exhibits a SPR in the red visible region, which can be useful for optical imaging, and presents a convenient surface for conjugating targeting moieties, while the iron oxide core provides a strong T_2_ (spin–spin relaxation time) MRI contrast. 

Li et al. [17] developed hyaluronic acid modified FANSs with a star morphology for MR imaging, thermal imaging and photothermal therapy of tumours. They prepared, first by way of the hydrothermal route, Fe_3_O_4_-Au core-satellite particles and used them as seeds to produce the final nanostar-shaped objects. Further sequential modifications of the resulting particles by polyethyleneimine (PEI) and hyaluronic acid afforded them an excellent colloidal stability, a good biocompatibility, and a targeting specificity to CD44 receptor-overexpressing cancer cells. 

Zhou et al. [8] reported the synthesis of Fe_3_O_4_-Au nanostar-shaped core-shell particles for targeting and multimodal imaging of cancer. They evidenced the potential of these NSs for a multimodal MRI, microwave-induced PA and PA imaging. Hu et al. [100] prepared also these anisotropic in shape FANSs but they successfully coated them with folic acid to make them targeted multimodal MR, CT and PA imaging probes.

### 4.2. Multimodal Therapy 

The use of heat has been emerged as a promising tool for cancer therapy. The heating is created from radio frequency, microwaves and ultrasound waves, and used in a specific target region, to perform hyperthermia and treat locally cancers. The cancerous cells being much more sensitive to the temperature increase than healthy ones, such an innovative approach allows selectively damaging and killing at a temperature ranging between 41 °C and 48 °C, the former instead of the later. The healthy cells can stand alive at so high temperatures. Recently, several novel developments based on the ability of NPs to generate heat under an external stimulus have demonstrated their potential in the treatment of cancers. Typically, the fluctuation of the magnetic moment of magnetic NPs exposed to an alternating magnetic field, transforms the electromagnetic energy into heat. This heat is introduced by the NP magnetic hysteresis loss, Brownian relaxation and Néel relaxation. The amount of generated heat depends on the size and the magnetic properties of NPs. For multi-domain ferri- or ferromagnetic NPs, the heat is only generated by the hysteresis losses. The orientation of the magnetic moments inside the material takes place through the displacements of the magnetic domain walls. For superparamagnetic single domain NPs, the heat is generated by Brownian and Néel relaxations. Brownian relaxations are related to the friction between the NPs and their fluidic media when they are submitted to an oscillating magnetic field. Néel relaxations are associated to the rotation of the magnetic moment with the oscillation of the field within each particle. 

Heat energy can be also generated by exposing metallic or dielectric NPs to a laser source, these NPs being able to absorb light, through their plasmonic [103,104] and phonon resonances [105,106], respectively, and to convert it in heat. NIR lasers can cross human tissues with minimal damages. The best material able to absorb in NIR region (650–1000 nm) for such an application is gold, due to its low cytotoxicity, its biocompatibility and strong optical absorbance in the NIR region. Besides, small gold particles can be aggregated and accumulated in tumours via a passive mechanism and can be used then as local photothermal sources. They can be also cleared out from the body as the therapy is complete, making them particularly valuable for in vivo applications. Very recent works have evidenced the ability of iron oxide to be used for photothermal therapy, exciting its lattice phonons around in the NIR region too, typically around 800 nm. This approach is just emerging and very few works exist in the literature to achieve a complete understood of the involved physical phenomena. 

Integrating heating capabilities, magnetic hyperthermia and photothermia in a unique object presents several advantages. For example, magneto-plasmonic nanostructures can be well bio-distributed into the body. They can be accumulated into the malignant tissues, generating heat very locally, when submitted to a light or a magnetic stimulus, preventing damages to healthy cells. Like for the optical imaging application, a fine-control of the morphology of the prepared FANSs and AFNSs is of primary importance, to promote NIR induced heating. Typically, gold nanostars, nanorods, nanoflowers offer strong absorption cross-sections. For magnetic hyperthermia, iron oxide multicore FANSs are much more recommended to reach high heating, due to their high magnetization. 

Espinosa et al. [14] combined successfully these two heating modalities using NPs composed of an iron oxide multicore particle coated by a gold shell with tunable plasmonic properties in NIR region. They typically observed an ultrafast tumour temperature increase up to 48 °C, a mandatory condition for therapeutic tumour destruction.

### 4.3. Biosensors and SERS Application

Surface enhanced Raman spectroscopy (SERS) has become one of the most important optical-based analytical techniques for the detection of chemical and biological molecular traces (Table 1). Well-shaped plasmonic particles enhance SERS signal, providing specific fingerprint information for molecular recognition at the single molecule level. With highly sensitive, selective and multiplexed detection capabilities, SERS can be used for in vivo and in vitro studies. Raman signals located on the surface of anisotropic in shape metallic nanostructures such as tips, edges and vertices can be enhanced by over 10 orders of magnitude. Therefore, SERS provides information even on traces of the target molecules. 

Recently, much effort has been developed to still improve SERS enhancement. One of the most important enhancement factors contributing to the SERS effect is the electromagnetic enhancement which emerges from SPR. The electromagnetic field surrounding plasmonic materials is not uniformly distributed. Its special distribution depends on the structure of the material such as the shape, size, aggregation state, etc. Strong electromagnetic fields could be highly localized in spatially narrow regions, such as nanotips, or a junction between adjacent plasmonic particles (hot spots). 

Among various plasmonic materials used for such a purpose, gold remains the most studied, because it can support SPR in the visible region. Its low excitation energy reduces the risk of analyte photobleaching and photodegradation. Its surface is stable toward oxidation. Gold nanomaterials with various size, shape and assembled on substrates have been thus reported. 

The integration of an iron oxide component to these gold structures is now receiving a great attention. In one hand, the dielectric properties of the oxide affect the SPR Au response, and in the other hand its magnetic properties can be used for separation and/or pre-concentration of the analytes before SERS application. FANSs, in majority, and AFNSs, in minority have been thus developed for such a purpose [107,108,109,110,111]. They possess the advantages of convenient binding to biomolecules and easy separation using a magnetic field. They can be used for detection of various biological molecules such as proteins, nucleic acids, antibody, cells, hormones [112]. They can be served as carrier for protein or many enzyme molecules. The conjugates of nanostructures with the antibody can be then collected by a magnetic field and deposited on an adapted substrate to be used as SERS-based sensor [113,114].

In practice, SERS applications require magneto-plasmonic hetero-nanostructures in two configurations: (i) colloidal suspensions and (ii) particles immobilized on a substrate. The preparation of colloidal suspensions for SERS experiments is the simplest approach for a good stability and high SERS performance. SERS measurements are easy to achieve, since the engineered NSs have only to be mixed to the analytical solution. A first spectrum can be recorded on a solution very diluted in target molecules. A second spectrum can be achieved on the sample after having applied an external magnetic field for enriching and separating the analytes from the solution, improving thus the detection limit. A monolayer of NSs at the interface of two non-miscible solutions can also be envisaged for SERS detection [29,42]. For example, a monolayer of FANSs at the hexane-water interface was successfully assembled and an external magnetic field was applied to balance the electrostatic interactions, surface tensions and magnetic forces, leading to the expansion and shrinkage of NS distances. Therefore, SERS measurements can be modulated thanks to the dynamic response of the interparticle spacing [45]. 

The magnetic field can also be used to immobilize NPs on a solid substrate. In this case, the field induces the formation of aggregated or multi-layered structures and facilitates the cyclic use of the NSs. Graphene and graphene-derived materials, which exhibit excellent electronic properties, are considered as suitable substrates for such an application. Graphene can reduce the SERS background, improving thus the Raman signal to noise ratio. Graphene oxide possesses active oxygen sites and highly negative surface charge, enhancing the absorption of positively charged NSs [37,115].

## 5. Conclusions

This work provides an overview of the relationships between the synthesis, the structure and the properties of multifunctional magneto-plasmonic nanomaterials, based on sequentially architected iron oxide and gold or reversely gold and iron oxide NSs. It also focuses on their applications in the biomedical field. The combination of magnetic and optical properties of gold and iron oxide offers a great potential for magnetic and optical imaging, hyperthermia and photothermia therapy, SERS detection and so on. Besides, from a materials science point of view, these magneto-plasmonic NSs usually exhibit good biocompatibility and colloidal stability. Their physical and chemical properties can be easily tuned by simply acting on their morphology and surface functionalization, respectively. Various morphologies of multifunctional NSs have already been developed, including spherical and non-spherical core-satellite and core-shell particles, leading to interesting magnetic and optical properties. Anisotropic in shape particles have been obtained by controlling the synthesis parameters. Many synthesis methods were used, from simple “one-pot” to complex “multi-step” ones, from seed-mediated to non-seed-mediated routes, adding or not surfactants etc. All these methods present some advantages and challenges to be solved. The engineered NSs generally present the same magnetic properties than their Fe_3−*x*_O_4_ component alone, with a decrease of the saturation magnetization because of the mass contribution of Au. They also exhibit SPR feature in a wavelength window ranging from the visible to the NIR spectral region depending on their size and shape. Most of the studies have been focused on anisotropic NSs because they possess the chemical, physical, biological properties that are difficult to achieve by spherical ones, in order to use them in multimodal medical therapy and diagnostic. 

## Figures and Tables

**Table 1:** FANSs and AFNSs for SERS applications.

Core Material	Shell Material	Reducing Agent	Application	Ref.
Fe_3_O_4_-SiO_2_-NH_2_ 175 nm	Au 185 nm	Formaldehyde	Detection of rhodamine	[10]
Fe_3_O_4_-BPEI 380 nm	Au 420 nm	Electrostatic interaction	Detection of low abundance of cancer cell	[44]
Fe_3_O_4_-PEI 40 ± 2 nm	Fe_3_O_4_@Au 140 ± 5 nm	NH_2_OH·HCl	Detection of free PSA	[116]
Fe_3_O_4_ 33 ± 9 nm	Star-shaped Au 146 ± 48 nm	DMF with PVP	Detection of dye molecule of Astra blue and protein magnetic separation	[13]
Fe_3_O_4_ 16.2 ± 2.8 nm	Star-shaped Au 80 nm	DMF with PVP	Detection of crystal violet	[22]
Fe_3_O_4_-PEI 15 nm	Star-shaped Au 248 ± 36 nm	NH_2_OH	Detection of bacteria	[21]
Fe_3_O_4_-PEI 200 nm	Au 220 nm	NH_2_OH·HCl	Detection of free-Prostate specific antigen, MRI, magnetic hyperthermia	[117]
Fe_3_O_4_-SiO_2_-NH_2_ 126 ± 15 nm	Star-shaped Au 160 ± 10–200 ± 16 nm	Formaldehyde; AgNO_3_	Detection of cancer-related miRNA	[61]
Au 90 nm	Fe_3−*x*_O_4_ 97 nm	TREG	Analysis of the structure and the chemical composition of the surrounding Fe_3−*x*_O_4_ shell	[11]

## Glossary

NPs	nanoparticles
CSNPS	core-shell nanoparticles
MRI	magnetic resonance imaging
NIR	near-infrared region
NSs	nanostructures
QDs	quantum dots
DOX	doxorubicin
FANSs	Fe_3−*x*_O_4_-Au nanostructures
AFNSs	Au-Fe_3−*x*_O_4_ nanostructures
TEM	transmission electron microscopy
PVP	polyvynilpyrolidone
PSA	serum albumin protein
DMF	*N*,*N*-dimethylformamide
APTMS	3-aminopropyltrimethoxysilane
miRNA	micro ribonucleic acid
PEI	polyethylene imine
SPR	surface plasmon resonance
NDs	nanodumbbells
RF	Radio Frequency
DFM	dark field microscopy
OCT	optical coherence tomography
CT	computed tomography
PA	photoacoustic
SERS	surface enhanced Raman scattering
TREG	triethyleneglycol
UV-Vis	ultraviolet-visible
FC	field cooling
ZFC	zero field cooling
